# Effect of Beta-Lactam Antibiotics on Microspore Embryogenesis in *Brassica* Species

**DOI:** 10.3390/plants9040489

**Published:** 2020-04-10

**Authors:** Anna Mineykina, Daria Shumilina, Ludmila Bondareva, Alexey Soldatenko, Elena Domblides

**Affiliations:** Laboratory of Reproductive Biotechnology in Crop Breeding, Federal State Budgetary Scientific Institution Federal Scientific Vegetable Center (FSBSI), Selectsionnaya str., 14, Odintsovo district, 143072 Moscow region, Russia; dasha2409@yandex.ru (D.S.);

**Keywords:** antibiotics, ampicillin, cefotaxime, in-vitro microspore culture, embryo development, embryogenesis induction, *B. oleracea*, *B. napus*, *B. rapa*

## Abstract

Antibiotics are widely applied for plant cultivation in vitro to eliminate bacterial contamination. However, they can have both positive and negative effects on the cells of cultivated plants, and these effects largely depend on the type antibiotic used and its concentration. The objective of the present study was to estimate the effect of β-lactam antibiotics ampicillin (Amp) and cefotaxime (Cef) on microspore embryogenesis induction in vitro in the *Brassica* species. The performed experiments confirmed cefotaxime inhibits microspores in *B. napus* and *B. oleracea*, even in concentrations as low as 50 mg/L. The highest embryo yield was obtained for *B. napus* in the NLN-13 medium with added ampicillin in concentrations of 50–100 mg/L as an antimicrobial agent. This embryo yield was significantly higher than that obtained in a medium without supplemented antibiotics and two times higher than in the medium with added cefotaxime. Analogous results were obtained for *B. oleracea* and *B. rapa*.

## 1. Introduction

Double haploid (DH) production has become one of the main tools of advanced plant breeding [1]. The isolated microspore culture (IMC) method is based on microspores changing their gametophytic to a sporophytic one when affected by stress factors such as increased temperature, high osmotic pressure, etc. Under certain conditions, such spores start developing a sporophytic pathway, forming embryos [2]. This method accelerates obtaining homozygous starting material (DH-lines) for breeding and widening the spectrum of genetic recombinant forms, including those with recessive traits. Although obtaining stable homozygotic lines from a population facilitates searching for rare genotypes, their embryogenic development depends on a variety of factors, the most important of which are the genotype, donor plant growing conditions, timely bud harvesting, as well as stress and environmental and cultivation conditions [3]. For in vitro culture, both endogenic and exogenic bacterial contamination are regarded as some of the main problems in androgenesis induction that may lead to losing some of the rare and precious samples. The source of contamination can be infected donor plants or improper septic conditions during manipulations in a laminar flow cabinet [4,5,6,7].

To mitigate or completely eliminate such sources of contamination, a range of widely known antibiotics can be added into a culture medium [8,9,10]. Despite their successful application, antibiotics can be phytotoxic and have different effects on plant embryogenesis and regenerative ability [11,12,13]. Multiple studies in different cultures demonstrated that antibiotics can both inhibit the growth and development of explants and cells and induce their regeneration [14,15,16,17,18,19,20]. It is assumed the stimulating effect is due to the auxin-like structure of β-lactam antibiotics that enables them to imitate the effect of plant hormones [21,22]. This is a large group of antibiotics with a β-lactam ring in their structure and a bactericidal effect that includes penicillins, cephalosporins, carbapenems, and monobactams. Their similar chemical structures predetermine their similar bactericidal mechanism that destroys the synthesis of bacterial cellular walls. Their effect on different cultures in vitro has been demonstrated in rice (*Oryza sativa* L.), carrot (*Daucus carota* L.), Chinese cabbage (*B. chinensis* L.), and others [13,17,21].

The objective of this study was to investigate the effect of the aforementioned antibiotics on the embryogenesis of a microspore culture in vitro for some species of the *Brassica* plants that are widely used in the world.

## 2. Results

**Figure 1:**
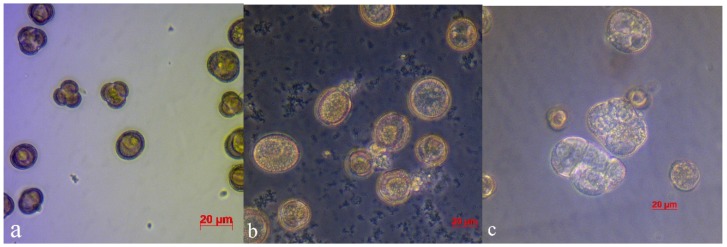
Culture of *B. napus* microspores: (**a**) in a medium without antibiotics after 1-day cultivation; (**b**) microspore development in medium without antibiotics after 5 days cultivation with traces of bacterial contamination; and (**c**) division of microspores in medium with ampicillin on day 5 day of cultivation.

**Figure 2:**
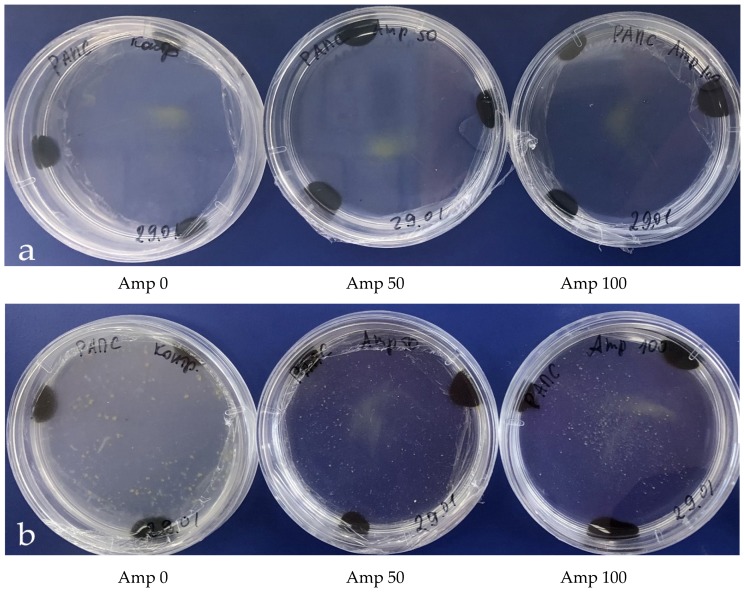
The development of embryos in in vitro microspore culture in *B.* *napus*: (**a**) first day of cultivation; (**b**) fifth day of cultivation (development of bacteria in media without antibiotics and absent any contamination in medium with ampicillin).

**Figure 3:**
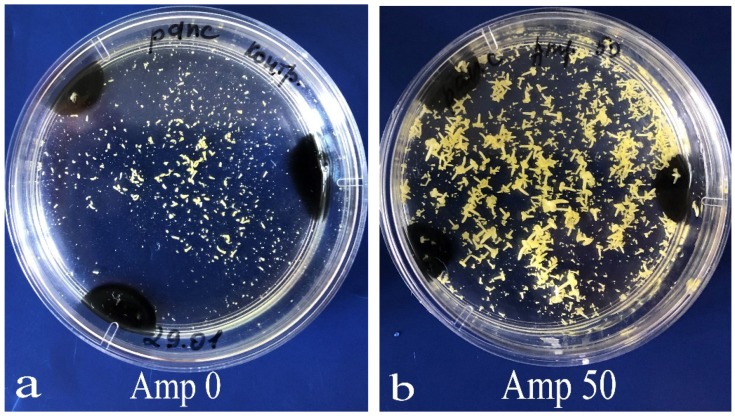
Petri dishes with embryos *B. napus* on 21 days in a medium (**a**) without antibiotics (embryo development was delayed, perhaps because of bacteria contaminations) and (**b**) with ampicillin (Amp) at a concentration of 50 mg/L.

When grown out-of-doors, the starting material seriously suffered from endogenic bacteria, which quickly reproduced at the stage of microspore division, suppressing embryogenesis, resulting in loss of up to 100% of the material. When microspores were cultivated in a medium without added antibiotics, developmental scenarios observed in the Petri dishes were as follows: (a) active bacterial growth accompanied by destruction of microspores (Figure 1b), the medium appeared turbid at five days (Figure 2b); (b) weak bacterial growth seemingly due to weak phytotoxic effect, microspore embryo formation was still observed, although inhibited and reduced in amount (Figure 3a); and (c) normal microspore embryo development in large amounts (Figure 3b).

A suspension of fresh isolated microspores of *B. napus* was used for inoculation of LB (Lysogeny Broth) bacterial growth media with and without added antibiotics to determine the efficacy of the antibiotics for bacterial growth suppression.

**Figure 4:**
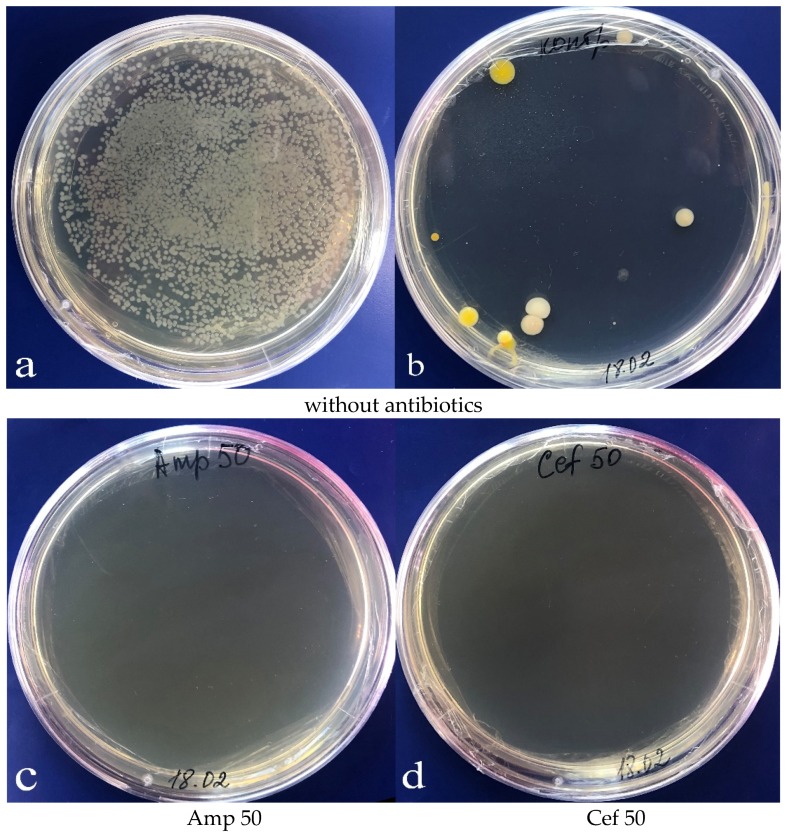
Determination of the sensitivity of contaminating bacteria in microspore culture to antibiotics on LB medium: (**a**) control development of bacteria colony with similar phenotype on LB medium without antibiotics; (**b**) control development of phenotypic diversity bacteria colony on medium without antibiotics; (**c**) inhibition of the bacteria growth on LB medium with ampicillin 50 mg/L; (**d**) inhibition of the bacteria growth on LB medium with cefotaxime 50 mg/L.

Then, the growth of bacterial colonies in the LB medium was observed (Figure 4a); yet, in some cases, no bacteria were present in the microspore culture. Notably, the composition of bacteria emerging in the *B. napus* microspore culture was rather diverse in terms of exterior morphological characteristics (Figure 4b). Bacterial growth was suppressed in the LB medium, when an antibiotic, either cefotaxime or ampicillin, was added in concentrations of 50 mg/L and above (Figure 4c,d).

**Figure 5:**
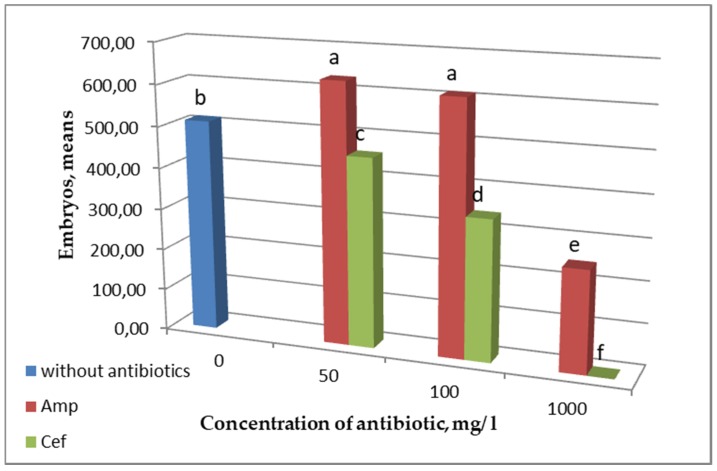
Effect of antibiotic type and concentration on *B. napus* embryo yield. Values marked with similar letters had no significant differences at *p* ≤ 0.05.

To determine the efficacy of antibiotics in a culture medium, we compared how antibiotic type and concentration affected the mean *B. napus* embryo yield (Figure 5).

It was shown that adding ampicillin led to embryogenesis induction in the *B. napus* microspore culture (Figure 5). The embryo number in the medium with antibiotic concentrations of 50 and 100 mg/L statistically exceeded that in the control medium without antibiotic. Increasing the concentration up to 1000 mg/L inhibited embryogenesis induction significantly.

Unlike ampicillin, cefotaxime inhibited embryogenesis induction even at a low concentration, whereas at a concentration of 1000 mg/L, microspore development was suppressed completely.

The next stage of the investigation was to determine the effect of antibiotics at a concentration of 100 mg/L on a microspore culture embryogenesis in various *Brassica* species. The concentration of 100 mg/L was chosen to ensure the most active bacterial suppression, according to the literature [15,21,23].

A series of experiments to determine the effects of ampicillin and cefotaxime on the embryogenic activity of an in vitro microspore culture in the *Brassica* plants demonstrated that the antibiotics affected the embryo yield in different ways (Table 1).

In genotype 127 of *B. oleracea*, the highest embryogenic activity was observed in the medium with ampicillin, whose embryo yield was 275.3 ± 17.4 per Petri dish, which was 1.5 times higher than that of the control medium. In the medium with cefotaxime, this embryo yield reduced five times relative to the control.

Genotype 145 of *B. oleracea* proved to be less responsive, and its control embryo yield was 33.3 ± 12.6 per Petri dish. Adding cefotaxime changed little, whereas in the medium with ampicillin, the embryo yield increased a number of times. Among all the species in the study, *B. rapa* was the least responsive: in the control medium, its embryo yield did not exceed 3.20 ± 0.04 per Petri dish. Adding ampicillin improved the situation and increased the embryo yield to 8.60 ± 0.17 per Petri dish.

**Figure 6:**
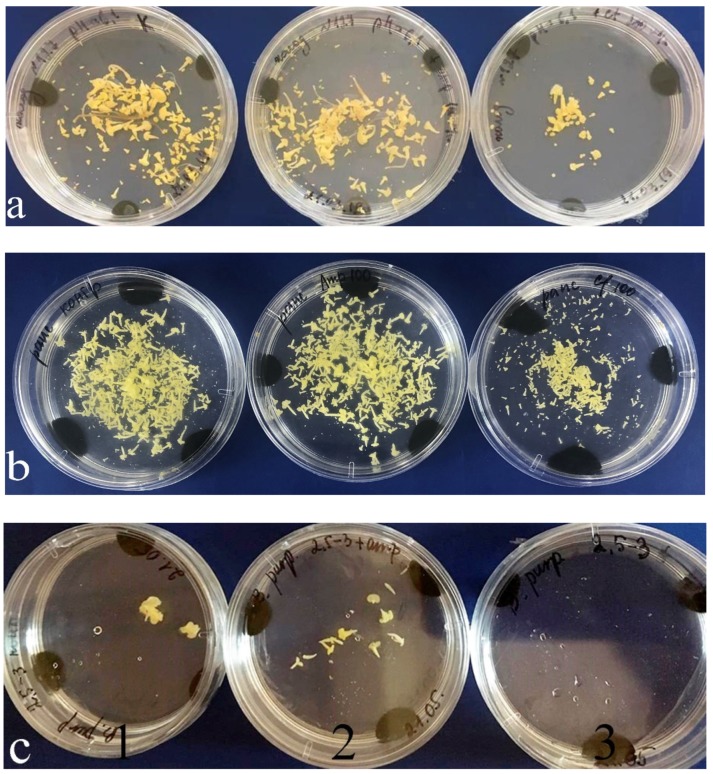
Effect of antibiotics on the embryogenic activity of the *Brassica* plants in in-vitro culture: (**a**) *B. oleracea;* (**b**) *B. napus*; (**c**) *B. rapa*. 1, control medium without antibiotics; 2, medium with ampicillin; 3, medium with cefotaxime.

Both in the control medium and in the medium with ampicillin, embryos developed much faster than in the presence of cefotaxime. They also reached the cotyledonary stage faster, that is, on the 28th day. In the medium with cefotaxime, only singular embryos reached this stage; the rest remained at globular and torpedo stages (Figure 6).

## 3. Discussion

In the present study, we used surface-sterilized plant material from in vitro isolated microspore cultures of *Brassica* species to observe different scenarios of microspore development. A standard sterilization procedure was sufficient for effective elimination of bacteria and ensuring normal microspore development, when the amount of initial bacterial infection at the surface of plant material was small (e.g., in the young plants grown in artificial climate chambers). Strong bacterial contamination of plant material or improper septic conditions produced drastically different results. In this case, bacterial growth was observed in the medium, whereas microspore development was suppressed completely. We also encountered a case where no signs of bacterial contamination were observed but embryo development was inhibited due to a hidden infection (Figure 3a). The use of antibiotics is a common solution for the bacterial contamination problem, and, according to the literature, β-lactam antibiotics are those most widely used in plants. Their bactericidal effect involves preventing the synthesis of peptidoglycan, which is the main component of bacterial cellular walls, by inactivation of transpeptidase on the inner surface of bacterial cellular membranes. In microbiology, medicine, and biology, ampicillin is generally used at a concentration of 100 mg/L to prevent bacterial contamination. Cefotaxime is used rather often for plant cell cultures and somatic embryogenesis in concentrations dependent on the culture and presence of bacteria. The concentrations vary from 50 to, for example, 500 mg/L for suppressing agrobacterium growth after genetic transformation of plant tissue [13,15,19,21,23,24,25,26,27]. The experiments with inoculation of microspore suspension in Petri dishes with LB bacterial growth medium performed in the present study showed that the presence of antibiotics ampicillin and cefotaxime at of 50 mg/L in the LB medium was sufficient for suppression of the bacterial contained in the microspore culture. In a medium without antibiotics, bacterial colonies of various appearances emerged. Identifying the composition of bacterial species was beyond the scope of the present study, although similar studies were performed by other researchers, showing that 50 mg/L cefotaxime suppressed many of the studied bacteria in triticale microspore culture [28]. In the present investigation, active antibiotic concentrations determined for the LB medium were then applied to a microspore culture. They were shown to effectively reduce contamination during embryo formation.

In addition to high antibacterial performance in a culture medium, an antibiotic should be devoid of negative effects on embryo and morphogenesis in plant cells and tissue. To the best of our knowledge, the studies on the effects of β-lactam antibiotics mostly considered somatic embryo formation. Despite their wide use for different biotechnological applications, antibiotics in microspore culture in vitro is beyond the standard protocol of obtaining DH-lines via androgenesis. The published literature contains only singular descriptions of such experiments in wheat, triticale, and rapeseed [27,28].

Meng et al. [17] confirmed the positive effect of ampicillin on the somatic embryogenesis of *B. rapa*. They noted that the use of 1000 mg/L ampicillin increased shoot differentiation frequency relative to the control medium without antibiotics.

Asif et al. [28] described the stimulating effect of 100 mg/L cefotaxime on wheat and triticale embryogenesis in microspore culture in vitro. Other authors demonstrated its inhibiting effect on *B. napus* embryogenesis and regeneration if the treatment has the required concentration and duration [27]. In the present study, the effect of antibiotics at concentrations of 50, 100, and 1000 mg/L on embryo formation was tested. For control, Petri dishes with microspore culture without antibiotics were used, but if during the experiment any bacterial contamination was observed, such Petri dishes were deleted from counting, which was necessary for understanding the influence of antibiotics on the microspore without bacterial contaminations. Cefotaxime has a similar inhibiting effect as *B. napus* embryogenesis on the embryogenesis of other *Brassica* plants even at concentrations as low as 50 mg/L.

In our study, 50 to 100 mg/L ampicillin had a stronger effect on embryo yield not only if compared with cefotaxime but also with a medium without antibiotic treatment, which is in agreement with the experimental data obtained for ampicillin and its effect in tomato and Chinese cabbage tissue cultures [17,19]. The increased embryogenesis and regeneration may be due to ampicillin’s nontoxicity for plant cells and tissues and its auxin-like structure. However, the mechanism of the effect remains unknown. The use of ampicillin at a high concentration of 1000 mg/L had an inhibitory effect on embryo formation, contrary to the results of Meng et al. [17], where this ampicillin concentration in the medium was optimal for in vitro cultured cotyledons. The use of cefotaxime at such a high concentration resulted in a complete loss of embryogenic activity in *B. napus* microspores.

The obtained results indicated that ampicillin is a preferable antibiotic for in vitro microspore cultivation of *Brassica* plants, since it effectively suppresses bacterial growth even in low concentration and does not inhibit embryo formation from microspores.

## 4. Materials and Methods

### 4.1. Growing the Donor Plants

In the study, the initial donor material was rapeseed (*Brassica napus* var. *napus*), white cabbage (*Brassica oleracea* L. convar. *capitata* (L.)), and Chinese zicaitai cabbage (*Brassica rapa* L. ssp. *Chinensis* Hanelt var. Hanelt *purpuraria* Kitam).

The donor plants were grown in an artificial-climate chamber at a temperature of 16–18 °C. The chamber worked in a 16 h day/8 h night mode and provided illumination of 9000 lx.

### 4.2. Microspore Development Stages

When the plants started blooming, their buds were harvested for cytological determination of microspore development stages. Differential staining [29] was performed to visualize the microspores, so a dependence between the bud size and microspore development stage could be determined using an Axio Imager A2 microscope (Zeiss, Dusseldorf, Germany). Bud size was picked depending on microspores development stages corresponding to late uninuclear to early binuclear pollen grains (30). Bud sizes were 2.5–3.5 mm for *B. napus*, 4.0–4.5 mm for *B. oleracea*, and 2.5–3.0 mm for *B. rapa*.

### 4.3. Microspore Isolation

The trial establishment was carried out in accordance with the guidelines on how to grow regenerant plants using isolated microspore culture in vitro [30] with minor modifications. The harvested buds, whose size corresponded to microspore development stages (from late uninuclear to early binuclear), were sterilized first in ethanol (70%) for 1 min and then in a 50/50 water solution of the Belizna bleacher (NaClO 2.5 %) (Belizna, Soltsy, Russia) mixed with Tween 20 (Panreac, Barselona, Spain) in proportion of 1 drop per 100 mL for 10 min. The sterilized buds were then rinsed three times in sterile distilled water for 10 min. The sterile buds were crushed in 13% sucrose NLN medium [31] (30 buds per 6 mL of medium). The slurry was filtered through a 40 micron nylon filter and centrifuged for 5 min in Eppendorf 5804R (Eppendorf, Hamburg, Germany) at 920 g. The microspores were rinsed twice in the NLN-13 medium. For each cultivar of the experimental plant, the microspore suspension density was determined using a hemocytometer (Precicolor Lutzellinden, Germany). For incubation, a microspore suspension of density 2 × 10^4^ mL^−1^ was used.

### 4.4. Bacterial Susceptible to Antibiotics

We used antibiotics ampicillin (Biosintez, Penza, Russia) and cefotaxime (Sintez, Kurgan, Russia) in lyophilized injectable form in sterile 1 g vials. To prepare the initial solution, sterile water (10 mL) was added to the sterile antibiotic (1 g); the solution was aliquoted by 1 mL and stored at −20 °C before use. Antibiotics were added to the sterile medium in accordance with the experiment design.

Sterile ampicillin and cefotaxime solution stocks were added to the LB medium before spilling into Petri dishes with a diameter of 9 cm to obtain final concentrations of 50, 100, and 1000 mg/L. As a control, Petri dishes with medium without the addition of any antibiotic were used.

The sterile solid LB medium was inoculated with 100 µL of freshly isolated microspores in a NLN-13 medium. Each experiment was performed two to three times in three different replicates (three Petri dishes for each variant). Petri dishes were cultured at 25 °C in the dark and bacterial colonies were checked daily. Most colonies grew after 24–48 h of incubation.

### 4.5. In Vitro Microspore Cultivation

Five mL of a microspore suspension (microspore isolation 4.3) in the NLN-13 medium was placed in a Petri dish with a diameter of 6 mm. Before placing a microspore suspension, we added 3–4 drops of sterile agarose solution of activated charcoal (Sigma-Aldrich, St. Louis, MO, USA) melted in a microwave (1 g activated charcoal per 100 mL of 0.5% agarose solution) in each Petri dish. After solidification of agarose with activated carbon, a suspension of microspores and antibiotic effluents was added to Petri dishes until the final concentration of 50, 100, or 1000 mg/L. As a control, there were dishes to which antibiotics were not added.

The dishes were incubated in darkness for 2 days at 32 °C and then continuously at 25 °C about 21 days when the embryos formed. For each of the genotypes, at least three independent experiments were performed in four replications for each variation. To analyze the effect of antibiotics to the development of microspores in embryos, we considered experiments where the control did not show the development of bacteria and inhibition of the development of embryos.

### 4.6. Statistic Analysis

Statistical analysis was performed using one-way analysis of variance (ANOVA) and factorial ANOVA, and means were compared using Duncan’s multiple range test (DMRT) with a probability of 95%. The statistical analyses were carried out using Statistica 8.0 (Statsoft, www.statsoft.com).

## 5. Conclusions

The experiments demonstrated that ampicillin and cefotaxime have different effects on the embryogenic activity of *Brassica* plants in microspore culture in vitro. Apart from its antibacterial effect, ampicillin was more effective than cefotaxime in stimulating embryo formation and development.

## Figures and Tables

**Table 1:** Effect of antibiotics (100 mg/L) added into a culture medium on the embryogenic activity of the *Brassica* plants in isolated microspore culture in vitro.

Species	Genotype	Mean Number of Embryos Formed Petri Dish		
		Without Antibiotics	With Ampicillin	With Cefotaxime
*B. oleracea* L. *convar. capitata* (L.)	127	181.8 ± 14.6 ^b^	275.3 ± 17.4 ^a^	35.8 ± 1.7 ^d^
	145	33.3 ± 12.6 ^d^	120.0 ± 34.7 ^c^	40.0 ± 10.3 ^d^
*B. rapa* L. ssp. *Chinensis* var. *purpuraria*	2	3.20 ± 0.04 ^e^	8.60 ± 0.17 ^f^	0.33 ± 0.05 ^g^

Note: Values marked with similar letter had no significant differences at *p* ≤ 0.05.
